# Supplementary material for: Genetic Diversity and Antimicrobial Resistance of Extraintestinal E. coli Populations Pre- and Post-Antimicrobial Therapy on Broilers Affected by Colisepticemia
Source: Animals (Basel). 2023 Aug 11;13(16):2590. doi: 10.3390/ani13162590 (PMC10451725; doi:10.3390/ani13162590)
Supplement: Supplementary file 1 [file animals-13-02590-s001.zip › Supplementary material MDPI Pasquali/Table S1 MDPI - Pasquali.pdf]

Table S1 – Statistics of the 31 newly sequenced whole genomes of *E. coli*

Samples	First coverage	N° of contigs	N° of bp	MLST type	N 50
871-21	84.9	248	5404654	117	108696
871-22	115.3	197	5210822	140	119872
871-41	129.1	282	5394093	117	76048
871-42	95.1	498	5400629	7080	78514
871-46	110.7	346	5036204	1618	54765
916-43	103.9	335	5020009	359	58277
976-38	96.3	770	5238046	43	41898
976-50	108.4	410	5152293	746	53531
976-57	118.5	99	5033925	117	174567
1614-21	73.1	271	4981152	23	72865
1614-23	93.3	163	4849691	23	109358
1614-35	110.8	207	4952965	23	139578
1614-41	103.4	198	5003675	23	102707
1665-22	103.5	476	5532331	7080	73626
1665-24	94.6	662	5533085	48	40182
1665-47	65.2	436	5227382	1196	38940
1684-49	93.9	327	5100617	155	59742
1684-52	136.3	609	5153625	43	54308
1832-43	86.6	413	5240210	1196	48379
2750-25	86.1	322	5127502	117	56562
2750-29	105.5	421	5305131	295	67276
2750-32	90.7	205	5097766	117	112051
2750-48	119.1	314	5228132	10	65030
2750-49	86.3	2062	6986556	101	21316
2835-26	104.1	271	5248241	1485	86166
2835-32	92.3	225	5238028	295	100534
2835-44	60.4	262	5128506	295	50108
2835-47	93.3	383	5020557	2040	42818
2835-57	94.2	340	5470555	997	97640
2863-46	54.3	569	5162506	295	23145
2863-50	89.5	358	5260718	295	55527
