# Supplementary material for: Genetic Diversity and Antimicrobial Resistance of Extraintestinal E. coli Populations Pre- and Post-Antimicrobial Therapy on Broilers Affected by Colisepticemia
Source: Animals (Basel). 2023 Aug 11;13(16):2590. doi: 10.3390/ani13162590 (PMC10451725; doi:10.3390/ani13162590)
Supplement: Supplementary file 1 [file animals-13-02590-s001.zip › Supplementary material MDPI Pasquali/Table S2 MDPI - Pasquali.pdf]

Table S2 – Metadata of newly sequenced *E. coli* genomes of broiler isolates

Farm	Isolate ID	Day of sampling	Origin	O Serogroup	MLST-Type	FimH-Type
A	871-21	1	spleen	NT	ST117	H97
	871-22	1	spleen	O2	ST140	H15
	871-41	1	lung	NT	ST117	H97
	871-42	1	lung	NT	ST7080	H54
	871-46	1	lung	NT	ST1618	H9
	916-43	5	lung	NT	ST359	H35
	976-38	10	heart	NT	ST43	NT
	976-50	10	lung	O20	ST746	H1429
	976-57	10	brain	NT	ST117	H97
B	1614-21	1	spleen	O78	ST23	H35
	1614-23	1	spleen	O78	ST23	H35
	1614-35	1	heart	O78	ST23	H35
	1614-41	1	lung	O78	ST23	H35
	1665-22	5	spleen	NT	ST7080	H54
	1665-24	5	spleen	NT	ST48	H23
	1665-47	5	lung	NT	ST1196	H31
	1684-49	10	lung	NT	ST155	H32
	1684-52	10	brain	NT	ST43	NT
	1832-43	24	lung	NT	ST1196	H31
C	2750-25	1	pleen	NT	ST117	H97
	2750-29	1	spleen	NT	ST295	H38
	2750-32	1	heart	NT	ST117	H97
	2750-48	1	lung	NT	ST10	H866
	2750-49	1	lung	NT	ST101	H86
	2835-26	5	spleen	NT	ST1485	H58
	2835-32	5	heart	NT	ST295	H38
	2835-44	5	lung	NT	ST295	H38
	2835-47	5	lung	NT	ST2040	H38
	2835-57	5	brain	NT	ST997	H31
	2863-46	10	lung	NT	ST295	H38
	2863-50	10	lung	NT	ST295	H38
